# The complex relationship between the digital divide, social capital, and mental health among older adults: a multi-method path decomposition

**DOI:** 10.3389/fpsyg.2025.1670203

**Published:** 2025-11-10

**Authors:** Qiwei Feng, Ting Zhou, Changxi Chen, Xinbin Xia

**Affiliations:** School of Humanities and Management, Hunan University of Chinese Medicine, Changsha, Hunan, China

**Keywords:** digital divide, mental health, older adults, social capital, suppression effect, causal inference, China

## Abstract

**Background:**

Against the backdrop of converging population aging and digitalization trends, the impact of the digital divide on older adults’ mental health represents a paradox characterized by conflicting empirical findings.

**Methods:**

To address this paradox, this study employs a multi-method analytical strategy—comprising propensity score matching, panel fixed-effects models, and generalized structural equation modeling—utilizing nationally representative data from the China Family Panel Studies (CFPS) 2016–2022. This approach systematically decomposes the complex causal pathways connecting the digital divide, social capital, and mental health among older adults.

**Results:**

After rigorously controlling for self-selection bias and time-invariant individual heterogeneity, our findings initially demonstrate a robust null total effect of the digital divide on depressive symptoms among older adults [average treatment effect on the treated (ATT) = 0.02, *t* = 0.10, *p* > 0.1]. However, subsequent mechanism analysis reveals that this null effect represents a statistical artifact arising from a suppression effect. Specifically, a beneficial direct pathway (direct effect of physical access on depression: *β* = −0.052, *p* < 0.1) is offset by a detrimental indirect pathway, wherein higher-order “motivational access” undermines bridging social capital (effect on interpersonal relationships: *β* = −0.207, *p* < 0.001), which subsequently serves as a protective factor for mental health (effect on depression: *β* = −0.032, *p* < 0.1).

**Conclusion:**

This study empirically establishes that the digital divide functions as a double-edged sword for older adults’ mental health, with its net effect contingent upon the complex interplay between direct technological benefits and indirect social costs. These findings indicate that future digital inclusion policies must transcend the narrow focus on bridging physical access gaps to prioritize “empowering trust.” This objective can be realized through targeted digital literacy interventions that enable older adults to navigate technology safely, thereby advancing the broader policy objective of healthy aging.

## 1 Introduction

The contemporary world stands at the nexus of two transformative trends: accelerating population aging and the ubiquitous proliferation of digital technology. This convergence has generated a “double jeopardy” for older adults, who confront novel forms of exclusion emanating from an increasingly “digital-first” society (Seifert et al., 2021; Antonucci et al., 2017), compounding traditional challenges including physiological decline and contracting social networks (World Health Organization, 2024). The global COVID-19 pandemic dramatically accelerated this convergence, highlighting the critical role of digital technology for maintaining social connection while exacerbating the risks of exclusion for those left behind. This phenomenon has emerged as a pressing global concern. The promotion of mental health and wellbeing constitutes a fundamental component of the United Nations’ Sustainable Development Goal 3, while the World Health Organization (WHO) and World Bank consistently emphasize that the “digital divide” is metamorphosing into a critical “development divide” and “health divide” (WHO, 2021; World Bank, 2024), with older adults representing one of the most profoundly affected demographics (World Bank, 2024). This challenge proves particularly pronounced in China, where the population aged 60 and above has reached 297 million (National Working Commission on Aging of the Ministry of Civil Affairs, 2024), with depressive symptom prevalence ranging from 20% to 40% (Zhao et al., 2025). While digital technology presents unprecedented opportunities for older adults to sustain social connections (Sen et al., 2022), its inherent accessibility barriers and skill prerequisites simultaneously generate novel impediments (van Deursen and Helsper, 2015; Vercruyssen et al., 2023). Consequently, elucidating the mechanisms through which the digital divide influences older adults’ mental health has emerged as an imperative research priority.

Within academic and policy discourse, an enduring debate persists concerning digital technology’s impact on older adults’ mental health, with empirical evidence yielding ostensibly contradictory findings. Certain studies adopt an optimistic stance, positing that digital inclusion facilitates social connection maintenance and loneliness mitigation among older adults, thereby enhancing mental health outcomes (Sen et al., 2022). Conversely, substantial evidence illuminates potential risks. Among certain older adults, the “Digital Grey Divide,” arising from deficits in skills, confidence, and cognitive capabilities (van Deursen and Helsper, 2015; Millward, 2003; Diana et al., 2025), may precipitate fear and anxiety when encountering unfamiliar technology, constituting a direct threat to mental health (Seifert et al., 2021; McDonough, 2016; Barreda Gutiérrez et al., 2024). Most critically, empirical findings remain markedly inconsistent; several systematic reviews and meta-analyses have identified non-significant or contradictory effects of digital technology on older adults’ mental health (Zhang et al., 2022; Rosell et al., 2023; Liu et al., 2025; Nimrod, 2020). This inconsistency likely originates from pervasive self-selection bias in extant research—namely, that healthier and more affluent older adults demonstrate greater propensity for internet utilization. This recognition compels the present investigation to reorient from examining whether direct effects exist toward a more fundamental inquiry: “Through which indirect mechanisms does the digital divide influence mental health?”

To address this scholarly debate, this study advances the proposition that researchers must transcend conventional approaches that conceptualize the “digital divide” and “social capital” as monolithic constructs, instead developing a more sophisticated theoretical framework. This framework emerges from the synthesis and refinement of two foundational theoretical perspectives.

First, to construct a nuanced analytical framework, this study draws upon the evolving multi-dimensional theory of the digital divide. The theoretical trajectory clearly demonstrates a deepening academic understanding of digital inequality: initially, the digital divide was narrowly conceptualized as the “access divide” (first level), denoting disparities in material accessibility to physical devices and internet connectivity (Telecommunications and Administration, 1999). However, as technology proliferated, scholarly attention shifted toward the “skills divide” (second level), as articulated by Hargittai (2002) and colleagues, which emphasizes inequalities in the competencies and literacies requisite for effective technology utilization (Hargittai, 2002). More recently, as delineated by van Deursen and Helsper (2015), the theory has evolved to encompass a “benefits divide” or “outcomes divide” (third level), focusing on disparities in tangible benefits derived from differential usage patterns (van Deursen and Helsper, 2015). Collectively, this multi-level progression from “access” through “skills” to “benefits” underscores a fundamental insight: mere device ownership does not constitute digital inclusion; rather, the critical challenge resides in subsequent effective utilization and the transformation of usage into meaningful outcomes (van Deursen and Helsper, 2015). This logic is formally captured by van Dijk’s “Resources and Appropriation Theory,” which posits that digital inclusion is a dynamic process of successfully converting technological resources (e.g., access) into tangible benefits through a series of “appropriation” processes, such as skills and usage (van Dijk, 2020; van Dijk, 2005). van Dijk (2020) four-stage model, which proves particularly comprehensive, offers a specific operationalization of this appropriation process, systematically disaggregating it into four sequential phases: “motivation” (willingness and attitudes toward technology adoption), “physical access” (material access to devices and networks), “skills” (competencies for effective technology utilization), and “usage” (breadth and depth of actual application) (van Dijk, 2020). While this study endorses van Dijk’s theoretical insight, to construct a more parsimonious empirical framework aligned with the CFPS data, we operationalize the digital divide into a three-level model comprising three core dimensions derived directly from van Dijk’s framework. Following the theoretical sequence, these are: (Seifert et al., 2021) motivational access, which corresponds to the first stage of “motivation” defined above; (Antonucci et al., 2017) physical access; and (WHO, 2021) usage. In this framework, the “skills” dimension is conceptually proxied by our measure of motivational access, which, as operationalized through subjective perceptions of the internet’s importance, reflects an individual’s confidence and perceived self-efficacy.

Second, an extensive body of literature indicates that digital technology’s impact on mental health is frequently mediated rather than direct, operating through the reconfiguration of individuals’ social capital (Ellison et al., 2007; Bekalu et al., 2019), which is widely recognized as a critical bridge connecting digital engagement to mental health (Liu et al., 2025). However, conceptualizing social capital as a monolithic construct obscures its inherent complexity. Therefore, this study adopts Putnam’s classical distinction, differentiating social capital into two categories: bonding social capital (BSC), which encompasses strong, exclusive ties within homogeneous groups (e.g., family members, close friends, tight-knit neighborhoods) that primarily provide emotional support; and bridging social capital (BrSC), which comprises weaker ties spanning diverse social groups, facilitating access to novel information and opportunities (Putnam, 2000).

**Figure 1:**
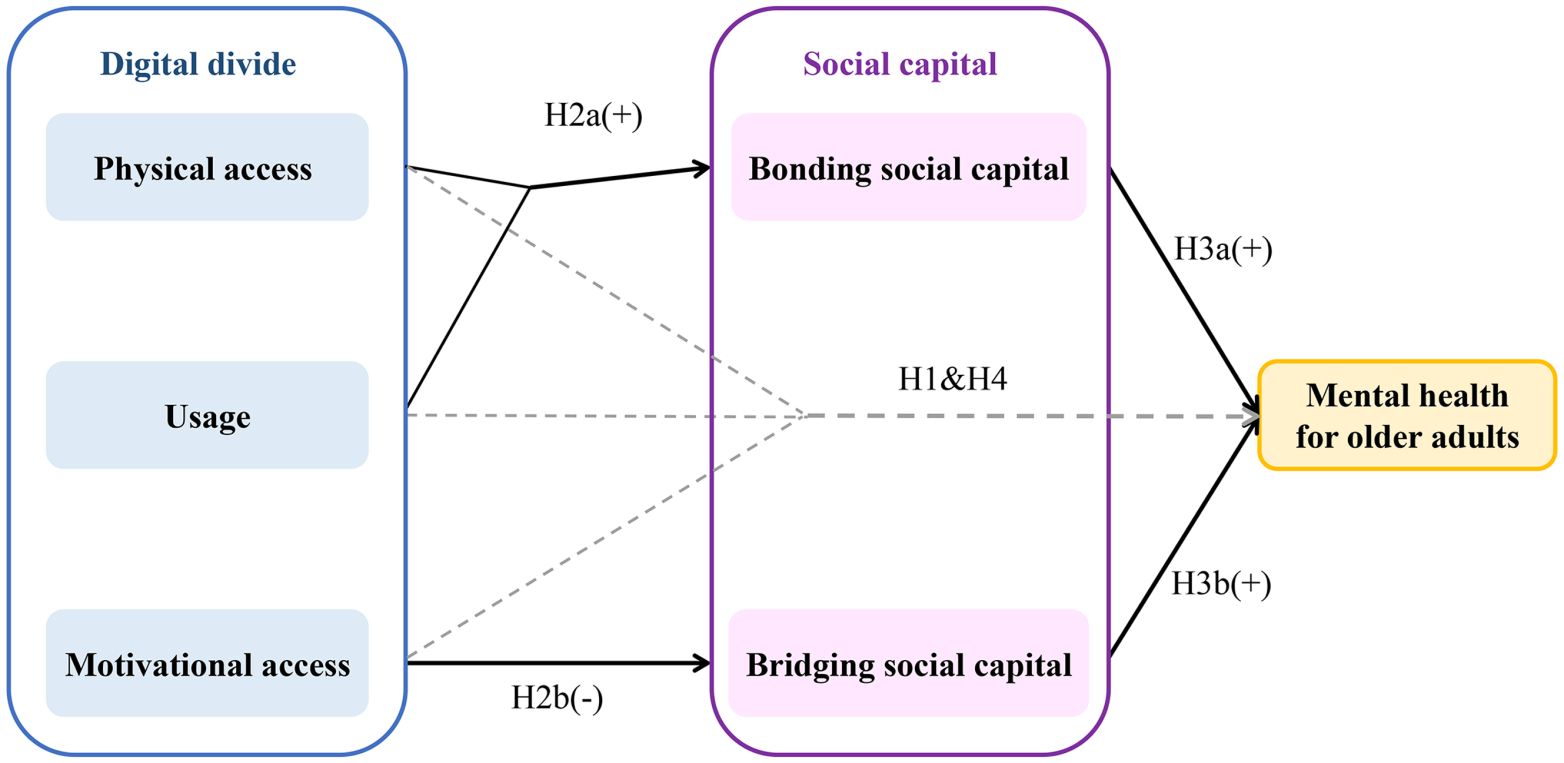
A conceptual model of the pathways linking the digital divide, social capital, and mental health in older adults. The model illustrates the core theoretical framework. Solid arrows represent the primary hypothesized mediating pathways (H2 and H3). Dashed arrows represent the direct pathways of the digital divide dimensions on mental health, which are hypothesized to be part of a complex suppression effect (H1 and H4). The “+” and “−” symbols indicate the hypothesized direction of the effect.

Building upon this theoretical foundation, this study advances its central theoretical contribution: the “Differentiated Social Capital Mediation Model” (Figure 1). The model’s fundamental premise posits that distinct dimensions of the digital divide exert differential effects on various social capital indicators, which in turn serve as mediating pathways to influence older adults’ mental health. We posit that these differentiated mediating pathways constitute the key to reconciling contradictions in extant literature. Specifically, we theorize that: (Seifert et al., 2021) basic physical access and elementary communication usage (e.g., video calls with family members) predominantly consolidate and reinforce existing BSC indicators; whereas (Antonucci et al., 2017) sophisticated digital competencies and elevated cognitive and motivational engagement represent prerequisites for establishing and sustaining BrSC indicators, as these necessitate the capacity and confidence to navigate heterogeneous online platforms and engage with individuals from diverse backgrounds. Conversely, skill deficiencies may undermine both the willingness and capacity to forge bridging ties. This mechanism suggests that mere “access” achievement may prove insufficient to generate anticipated mental health benefits and might precipitate a “bonding trap”—wherein older adults become confined within existing homogeneous social networks. Although familial ties may strengthen, broader societal connections may attenuate, consequently constraining opportunities to derive benefits from diverse social interactions.

Grounded in the aforementioned theoretical framework, this study advances the following core hypotheses:

*Hypothesis 1 (H1)*: After controlling for confounding factors, the direct total effect of the digital divide on older adults’ mental health demonstrates no statistical significance.

*Hypothesis 2 (H2)*: Different dimensions of the digital divide exert differentiated effects on various social capital indicators.

*H2a*: Physical access and usage positively influence BSC.

*H2b*: Motivational access negatively affects BrSC; specifically, diminished motivational access correlates with impaired BrSC.

*Hypothesis 3 (H3)*: Different social capital types exhibit differentiated roles and effect magnitudes in safeguarding older adults’ mental health.

*H3a*: As a fundamental source of emotional support, BSC exerts a primary and statistically significant protective effect against depressive symptoms.

*H3b*: While BrSC may confer protective benefits, its function relates more to provision of novel information and opportunities; its direct protective effect on mental health may prove secondary or manifest only within specific dimensions.

*Hypothesis 4 (H4)*: Social capital performs a critical and differentiated mediating function in the digital divide-mental health relationship among older adults. The total effect may exhibit a complex pattern of interaction between direct and indirect pathways, potentially encompassing suppression effects wherein pathways demonstrate opposing signs.

To address the limitations of previous research and rigorously examine the proposed theoretical framework, this study employs a multi-method analytical strategy leveraging complementary methodological strengths. The primary objective involves systematically decomposing the complex causal pathways connecting the digital divide, social capital, and older adults’ mental health through utilization of nationally representative longitudinal data from the China Family Panel Studies (CFPS) 2016–2022, integrating multiple econometric approaches including propensity score matching (PSM), panel fixed-effects (FE) models, and generalized structural equation modeling (GSEM).

This investigation contributes to the literature across three distinct dimensions, advancing theoretical perspectives and empirical evidence for comprehending older adults’ mental health in the digital era. Theoretically, it transcends conventional approaches that conceptualize the digital divide and social capital as monolithic constructs through the development of an innovative “Differentiated Social Capital Mediation Model.” Methodologically, it synthesizes three sophisticated econometric techniques (PSM, FE models, and GSEM) to establish a rigorous analytical framework that integrates causal inference with mechanism exploration. Empirically, it delivers a comprehensive examination utilizing large-scale, nationally representative data from China.

## 2 Materials and methods

### 2.1 Data source and sample

This study utilizes data from the China Family Panel Studies (CFPS), a nationally representative longitudinal social survey. Guided by the evolution of the CFPS questionnaire and our specific analytical requirements, we constructed two complementary datasets. First, a 2016–2020 unbalanced panel dataset for longitudinal causal inference. This timeframe was determined by two factors: 2016 was selected as the start year as preceding waves lacked requisite key variables, while the panel concludes in 2020 to ensure the strict temporal consistency in measurement required by the FE model, which was disrupted by changes in the 2022 questionnaire. Second, a 2022 cross-sectional dataset for mechanism exploration. Although the aforementioned changes to key indicators made it incompatible with the panel data, these updated and richer indicators were uniquely suited for in-depth analysis via PSM and GSEM, enhancing the timeliness and policy relevance of the findings.

The analytical sample was delimited to adults aged 60 years and above. Following exclusion of cases with missing data on core variables, we addressed random missingness for selected variables through back-filling and multiple imputation procedures. The final effective sample for panel analysis comprises 18,553 observations (16,537 weighted), while the effective sample for the 2022 cross-sectional analysis encompasses 4,290 individuals (4,102 weighted). All statistical analyses incorporated the complex survey design of the CFPS through application of standardized survey weights and adjustment for primary sampling units and strata, thereby ensuring national representativeness of the findings. These substantial sample sizes provide sufficient statistical power to detect even small to medium-sized effects, lending greater confidence to our interpretation of non-significant findings.

### 2.2 Variables

#### 2.2.1 Dependent variable

The dependent variable of this study is mental health, operationalized through depressive symptoms. This construct was measured utilizing the 8-item Center for Epidemiologic Studies Depression Scale (CES-D-8). The total score was derived by summing the eight items from the CFPS following recalibration of original values to a 0–3 scale (Turvey et al., 1999). The CES-D-8 represents a well-established abbreviated version of the internationally recognized CES-D scale and has demonstrated robust reliability and validity for assessing depressive symptoms in large-scale social surveys; elevated scores indicate increased symptom severity (Radloff, 1977). The utilization of depressive symptoms as a core negative indicator of mental health among older adults aligns with definitions established by the WHO and prevailing scientific literature (Tengku Mohd et al., 2019; WHO, 2015).

#### 2.2.2 Core independent variable

Drawing upon the fundamental tenets of van Dijk (2020) multi-dimensional theory of the digital divide (van Dijk, 2020), this study conceptualizes the digital divide as a multifaceted construct. Given the distinct objectives of different analytical phases and variations in the CFPS questionnaire across years, we implemented targeted operationalization strategies for this construct.

For the PSM analysis utilizing 2022 cross-sectional data, we constructed a binary treatment variable designated digital divide (Table 1). This variable was developed through a two-stage *K*-means clustering strategy, with the detailed construction process delineated in Section 2.3.

**Table 1:** Variable assignment and descriptive statistics of the 2022 cross-sectional sample.

Variable	Assignment	*N*/mean	%/SE
Dependent variable			
Mental health		5.60	0.14
Core independent variables			
Digital divide			
No	0	1,101	26.85
Yes	1	3,001	73.15
Physical access			
No	0	2,697	65.75
Yes	1	1,405	34.25
Usage (min/day)		44.75	3.32
Motivational access			
Non-user	0	2,080	50.70
Low perception	1	896	21.85
High perception	2	1,126	27.44
Core mediating variables: social capital			
Bonding social capital			
Neighbor trust		7.09	0.06
Financial transfers (CNY/year)		2305.23	176.53
Child–parent relationship		4.25	0.03
Contact frequency		2.37	0.04
Communication frequency		2.63	0.04
Bridging social capital			
Trust in strangers		2.42	0.06
Interpersonal relationships			
Poor/average	0	1,345	32.78
Good	1	2,757	67.22
Control variables			
Sociodemographic characteristics			
Age (years)		68.45	0.20
Gender			
Female	0	1,954	47.65
Male	1	2,148	52.35
Ethnicity			
Han Chinese	1	3,842	93.66
Other	2	260	6.34
Marital status			
Divorced/widowed/single/cohabiting	0	673	16.40
Married	1	3,429	83.60
Number of children			
0	0	1,048	25.54
1	1	2,101	51.22
2 or more	2	953	23.24
Socioeconomic status			
Education			
Illiterate/semi-literate	1	1,429	34.84
Primary school	2	959	23.38
Lower secondary school	3	968	23.61
Upper secondary education and above	4	746	18.17
PCHI (CNY/year)		31920.40	3837.79
Residence			
Rural	0	1,852	45.16
Urban	1	2,250	54.84
Psychological attitudes			
Self-rated social status			
Very low	1	236	5.76
Low	2	372	9.06
Average	3	1,587	38.69
High	4	996	24.28
Very high	5	911	22.21
Life satisfaction			
Very dissatisfied	1	22	0.54
Dissatisfied	2	84	2.06
Neutral	3	707	17.24
Satisfied	4	1,300	31.68
Very satisfied	5	1,989	48.48
Confidence in future			
Very unconfident	1	56	1.36
Unconfident	2	106	2.58
Neutral	3	774	18.86
Confident	4	1,125	27.42
Very confident	5	2,042	49.79
Regional and institutional factors			
Region			
Eastern	1	1,754	42.75
Central	2	1,446	35.24
Western	3	903	22.00
Basic social medical insurance			
Not enrolled	0	332	8.10
URRBMI	1	3,013	73.45
UEBMI/government-funded medical care	2	757	18.45
Health status and behaviors			
Self-rated health			
Extremely healthy	1	442	10.76
Very healthy	2	450	10.98
Relatively healthy	3	1,603	39.09
Average	4	614	14.96
Unhealthy	5	993	24.21
Chronic disease			
Without	0	2,912	70.98
With	1	1,190	29.02
Exercise frequency			
Never/less than once a week	0	2,495	60.82
1–6 times a week	1	331	8.07
Daily	2	1,276	31.10
Total		4,102	100.00

SE, standard error; PCHI, *per capita* household income; CNY, Chinese yuan; UEBMI, urban employee basic medical insurance; URRBMI, urban and rural basic resident medical insurance.

For the GSEM, also employing 2022 data, we disaggregated the digital divide into three observable variables to examine its constituent dimensions: physical access (internet accessibility via mobile devices or computers), usage (daily online time measured in minutes), and motivational access (overall perception of the internet’s importance) (Table 1). We posit that motivational access, operationalized through subjective perceptions, constitutes a valid proxy for individuals’ underlying digital literacy and perceived benefits.

For the FE analysis utilizing 2016–2020 data, we constructed a composite variable also designated digital divide for consistency; however, its measurement relied exclusively on indicators that remained common and identically defined across all three waves to ensure temporal consistency (Tables 2, 3). Multiple versions of this variable were generated using *K*-means clustering to facilitate robustness checks.

**Table 2:** Variable assignment and descriptive statistics of the 2016–2020 panel sample.

Variable	Assignment	2016		2018		2020	
		*N*/mean	%/SE	*N*/mean	%/SE	*N*/mean	%/SE
Dependent variable							
Mental health		6.68	0.15	6.94	0.15	5.53	0.17
Core independent variables							
Physical access							
No	0	6,790	93.27	4,857	87.71	2,876	77.34
Yes	1	490	6.73	681	12.29	843	22.66
Usage (min/day)		7.02	1.16	12.84	1.57	28.17	2.88
Importance of TV							
Very unimportant	1	776	10.67	450	8.12	297	7.98
Unimportant	2	590	8.11	381	6.89	216	5.81
Neutral	3	1,622	22.29	1,155	20.85	729	19.61
Important	4	1,357	18.64	1,016	18.35	675	18.16
Very important	5	2,934	40.30	2,536	45.79	1,802	48.44
Importance of Internet							
Very unimportant	1	6,319	86.80	4,072	73.52	2,186	58.79
Unimportant	2	253	3.47	348	6.29	221	5.93
Neutral	3	321	4.41	489	8.82	460	12.36
Important	4	162	2.23	260	4.70	333	8.94
Very important	5	225	3.09	369	6.67	520	13.97
Importance of radio							
Very unimportant	1	5,195	71.36	3,343	60.37	1,829	49.18
Unimportant	2	532	7.31	542	9.78	347	9.33
Neutral	3	635	8.72	737	13.30	510	13.71
Important	4	352	4.83	328	5.93	392	10.54
Very important	5	567	7.79	588	10.62	641	17.24
Importance of SMS							
Very unimportant	1	5,655	77.67	3,394	61.29	1,703	45.80
Unimportant	2	602	8.27	523	9.45	446	11.99
Neutral	3	501	6.89	752	13.57	592	15.91
Important	4	254	3.50	414	7.47	392	10.54
Very important	5	268	3.68	455	8.22	586	15.76
Core mediating variables: social capital							
Neighbor trust							
Low (scores 0–5)	0	2,507	34.43	1,668	30.11	1,073	28.84
Middle (scores 6–7)	1	1,484	20.38	1,233	22.26	771	20.73
High (scores 8–10)	2	3,289	45.19	2,638	47.63	1,876	50.43
Financial transfers (CNY/year)		1219.06	109.71	2594.59	222.33	1880.73	195.50
Child–parent relationship		4.26	0.03	4.26	0.02	4.36	0.03
Contact frequency		3.67	0.07	2.53	0.04	2.62	0.04
Communication frequency		4.03	0.05	2.85	0.03	2.78	0.04
Trust in strangers							
Low (scores 0–4)	0	5,986	82.22	4,413	79.68	2,899	77.94
High (scores 5–10)	1	1,294	17.78	1,125	20.32	820	22.06
Control variables							
Sociodemographic characteristics							
Age (years)		68.98	0.17	68.51	0.17	69.14	0.25
Marital status							
Divorced/widowed/single/cohabiting	0	1,665	22.87	1,201	21.68	713	19.19
Married	1	5,615	77.13	4,337	78.32	3,006	80.81
Number of children							
0	0	2,448	33.63	1,870	33.77	1,245	33.48
1	1	3,616	49.67	2,699	48.73	1,796	48.30
2 or more	2	1,216	16.70	969	17.50	677	18.22
Socioeconomic status							
Education							
Illiterate/semi-literate	1	3,988	54.78	2,807	50.68	1,762	47.39
Primary school	2	1,338	18.37	1,081	19.52	633	17.03
Lower secondary school	3	958	13.16	848	15.31	556	14.95
Upper secondary education and above	4	996	13.69	802	14.49	767	20.63
PCHI (CNY/year)		19896.08	1189.77	26157.10	3179.28	26610.17	1726.43
Residence							
Rural	0	2,951	40.53	2,592	46.80	1,674	45.01
Urban	1	4,329	59.47	2,946	53.20	2,045	54.99
Psychological attitudes							
Self-rated social status							
Very low	1	819	11.25	382	6.90	168	4.51
Low	2	1,099	15.10	657	11.87	364	9.78
Average	3	2,981	40.95	2,061	37.22	1,375	36.98
High	4	1,403	19.27	1,200	21.66	975	26.20
Very high	5	978	13.44	1,238	22.35	838	22.53
Wellbeing index		−0.12	0.02	0.15	0.02	0.21	0.02
Regional and institutional factors							
Region							
Eastern	1	2,977	40.90	2,326	42.00	1,661	44.66
Central	2	2,380	32.69	1,806	32.62	1,200	32.27
Western	3	1,923	26.42	1,406	25.38	858	23.07
Basic social medical insurance							
Not enrolled	0	533	7.32	366	6.61	380	10.22
URRBMI	1	5,251	72.13	4,095	73.95	2,611	70.21
UEBMI/government-funded medical care	2	1,496	20.55	1,077	19.44	728	19.58
Health status and behaviors							
Self-rated health							
Extremely healthy	1	478	6.56	490	8.84	385	10.34
Very healthy	2	781	10.73	568	10.26	399	10.73
Relatively healthy	3	2,260	31.04	1,962	35.43	1,356	36.46
Average	4	1,759	24.16	946	17.09	696	18.71
Unhealthy	5	2,003	27.51	1,572	28.38	884	23.76
Chronic disease							
Without	0	5,067	69.60	3,911	70.62	2,675	71.93
With	1	2,213	30.40	1,627	29.38	1,044	28.07
Exercise frequency							
Never/less than once a week	0	4,024	55.28	2,478	44.75	2,437	65.54
1–6 times a week	1	460	6.31	597	10.78	272	7.31
Daily	2	2,796	38.41	2,463	44.47	1,010	27.16
Total		7,280		5,538		3,719	

SE, standard error; PCHI, *per capita* household income; CNY, Chinese yuan; UEBMI, urban employee basic medical insurance; URRBMI, urban and rural basic resident medical insurance.

**Table 3:** Results of cluster analysis for the digital divide (2016–2020 panel data).

Variable	Digital divide (*k* = 2)			Digital divide (*k* = 3)				Digital divide (*k* = 4)				
	Cluster 1 (without digital divide)	Cluster 2 (with digital divide)	*F*-statistic	Cluster 1 (without digital divide)	Cluster 2 (medium digital divide)	Cluster 3 (high digital divide)	*F*-statistic	Cluster 1 (without digital divide)	Cluster 2 (medium digital divide)	Cluster 3 (high digital divide)	Cluster 4 (very high digital divide)	*F*-statistic
Physical access	1.9	−0.36	41665.67***	2.18	−0.36	−0.33	33234.63***	2.63	−0.37	−0.36	−0.31	110000***
Usage	1.37	−0.26	10770.82***	1.59	−0.26	−0.25	6782.14***	1.91	−0.26	−0.26	−0.25	6430.64***
Importance of Internet	0.12	−0.02	42.49***	0.09	0.63	−1.18	17085.06***	−0.01	0.46	0.32	−1.78	8105.56***
Importance of radio	1.78	−0.33	27554.22***	1.76	−0.21	−0.41	9695.21***	1.58	0.42	−0.43	−0.42	5189.57***
Importance of SMS	0.63	−0.12	1283.54***	0.56	0.07	−0.37	779.76***	0.36	1.22	−0.51	−0.37	6406.37***
Importance of TV	1.17	−0.22	6606.11***	1.17	−0.05	−0.44	3209.14***	0.97	0.79	−0.43	−0.48	3886.04***
*N*	2,552	13,985		2,245	9,368	4,923		1,920	3,768	8,486	2,363	
%	15.43	84.57		13.58	56.65	29.77		11.61	22.78	51.32	14.29	

Values in the table represent the cluster centers (means) for each group. All input variables used for clustering were *z*-score standardized. The *F*-statistic tests for significant differences between the cluster centers. ***p< 0.001.

#### 2.2.3 Core mediating variables

This investigation conceptualizes social capital as the primary mediating variable. Drawing upon the seminal theories of Putnam (2000) and Lin (2002), we differentiate between two fundamental dimensions: bonding and bridging social capital.

In the GSEM, BSC is operationalized through five distinct observable indicators: neighbor trust (an 11-point scale ranging from 0 to 10, with higher scores indicating greater trust), child–parent relationship (self-rated quality of relationships with children), financial transfers (financial support received from non-co-resident relatives) (Lyu and Sun, 2021; Zhang and Zhao, 2024), contact frequency (frequency of in-person meetings with children), and communication frequency (frequency of alternative contact forms with children, e.g., phone, video calls). These indicators, particularly family interactions (Li and Zhou, 2021; Hwang et al., 2022) and neighbor trust (Lu and Wu, 2022; Ren et al., 2023), have been extensively validated in social capital research.

BrSC is operationalized through two principal observable indicators: trust in strangers (an 11-point scale ranging from 0 to 10, with higher scores indicating greater trust) (Ren et al., 2023) and interpersonal relationships (self-rated social relations). The former represents a well-established measure of bridging social capital (Putnam, 2000; Ren et al., 2023). Regarding the latter indicator, we acknowledge its function as a proxy for individuals’ social network breadth given CFPS data constraints. We explicitly recognize this measurement limitation and will interpret associated findings with appropriate caution in subsequent analyses and discussion.

#### 2.2.4 Control variables

The selection of control variables was informed by the WHO’s established Health Determinants framework (Solar and Irwin, 2010) and pertinent empirical literature (Diana et al., 2025; Chen et al., 2025; Feng et al., 2025). The objective was to incorporate key factors consistently demonstrated to influence the core relationships under investigation, while accounting for data availability constraints. These variables encompass: (Seifert et al., 2021) psychological attitudes (life satisfaction, confidence in the future, self-rated social status); (Antonucci et al., 2017) sociodemographic characteristics (age, gender, ethnicity, marital status, number of living children); (WHO, 2021) socioeconomic status (education, per capita household income, residence); (World Bank, 2024) health status and behaviors (self-rated health, chronic disease, exercise frequency); and (Zhao et al., 2025) regional and institutional factors (region, basic social medical insurance). Specific variables and their operationalization are detailed in Tables 1, 2.

Acknowledging that different analytical models possess distinct statistical assumptions and objectives, we implemented a flexible, model-specific approach to control variable treatment. Specifically, in the PSM analysis, all multi-level scales were incorporated as ordinal categorical variables to maximize information retention and capture non-linear relationships flexibly. In the GSEM, to streamline model complexity and treat effects as continuous, key psychological attitudes including life satisfaction, confidence in the future, and self-rated social status were standardized and incorporated as continuous variables. In the FE model, to mitigate multicollinearity, the two highly correlated psychological attitudes—life satisfaction and confidence in future—were standardized, averaged, and integrated into a single composite index (wellbeing index). For the core social capital indicators—trust in neighbors and strangers—we implemented different categorization schemes tailored to each dataset based on post-hoc multiple comparison tests, ensuring optimal measurement robustness within each analytical module.

### 2.3 Analytical strategy

For clarity in model presentation, the following abbreviations denote core variables throughout all subsequent equations: MH for mental health, DD for the digital divide, and SC for social capital.

#### 2.3.1 Step 1: identifying digital divide groups via *K*-means cluster analysis

To operationalize the multi-dimensional “digital divide” construct into a categorical variable for subsequent analyses, this investigation employed *K*-means clustering methodology (MacQueen, 1967). This approach empirically identifies naturally occurring groups within the data based on individuals’ physical access, usage, and motivational access, thereby circumventing the arbitrariness inherent in subjective classifications.

Clustering variable selection and processing varied by dataset. For panel data, we utilized temporally consistent indicators encompassing physical access, usage, and four motivational indicators of internet importance (Table 3). For cross-sectional data, we incorporated physical access, usage, and five motivational indicators (Table 4). All input variables underwent z-score standardization to eliminate scale-related influences. The optimal cluster number (*k*) was determined through integration of the Calinski–Harabasz (CH) index and the Elbow Method.

**Table 4:** Results of cluster analysis for the digital divide (2022 cross-sectional data).

Variable	Digital divide (*k* = 2)		
	Cluster 1 (without digital divide)	Cluster 2 (with digital divide)	*F*-statistic
Physical access	0.13	−0.15	40.68***
Usage	0.22	−0.25	114.6***
Importance of the internet for work	0.58	−0.68	1453.68***
Importance of the internet for leisure	0.6	−0.69	1334.27***
Importance of the internet for socializing	0.47	−0.55	743.77***
Importance of the internet for learning	0.63	−0.73	1924.11***
Importance of the internet for daily life	0.57	−0.63	1279.47***
*N*	1,101	921	
%	54.45	45.55	

Values in the table represent the cluster centers (means) for each group. All input variables used for clustering were *z*-score standardized. The *F*-statistic tests for significant differences between the cluster centers. ***p < 0.001.

When constructing the binary treatment variable for PSM analysis, we implemented a two-stage strategy. Initially, we performed clustering exclusively on the “internet user” subsample—based on their physical access, usage, and perceived internet importance—to classify them as either “high-efficiency users” (the “without digital divide” group) or “low-efficiency users” (the “with digital divide” user group). Subsequently, we consolidated “low-efficiency users” with “non-internet users” to constitute the treatment group (cluster = 1), designated as the “outcome-based digital divide” group. While acknowledging heterogeneity within this treatment group, this operationalization rests on clear theoretical foundations: our investigation emphasizes the final outcome of digital divide rather than its etiology. From an outcome perspective, both “non-users” (lacking access) and “low-efficiency users” (lacking skills) occupy comparable positions of inability to effectively transform digital technology into social and psychological capital. Therefore, comparing them collectively against “high-efficiency users” (the control group), who achieve this transformation, enables the most direct examination of the average treatment effect of this “outcome-based digital divide” on mental health. This approach’s robustness was confirmed through sensitivity analysis reported in Supplementary Table S1.

#### 2.3.2 Step 2: cross-sectional causal inference via PSM

To address the self-selection problem arising from observable variables, we initially employed PSM methodology to estimate the ATT of the digital divide (Rosenbaum and Rubin, 1983). The process begins by constructing a logit model that accounts for the complex survey design, using the binary variable cluster (indicating membership in the “with digital divide” group) as the dependent variable to estimate individual propensity scores (see Equation 1).


$$P({\mathrm{DD}}_{i}=1|X_{i})=\frac{e^{\alpha_{0}+\alpha \prime X_{i}}}{1+e^{\alpha_{0}+\alpha \prime X_{i}}}\;\;$$ (1)

The covariate vector $X_{i}$ encompasses all relevant control variables delineated in section 2.2, striving to satisfy the ignorability assumption for propensity score estimation to the maximum extent feasible.

Following implementation of various algorithms to match individuals based on propensity scores and confirming balance achievement through balance tests, we calculated the ATT (see Equation 2).


$$\mathrm{ATT}=E[Y_{i}(1)-Y_{i}(0)|D_{i}=1]\;$$ (2)

Here, $D_{i}=1$ indicates treatment group membership (the “with digital divide” group), while $Y_{i}(1)$ and $Y_{i}(0)$ represent potential depressive scores for individuals with and without treatment, respectively.

#### 2.3.3 Step 3: longitudinal causal inference via FE model

To address omitted variable bias emanating from time-invariant, unobservable individual heterogeneity, we additionally employed a two-way fixed-effects model utilizing 2016–2020 panel data (Wooldridge, 2010). The model specification appears as Equation 3:


$$MH_{\mathrm{it}}=\beta_{1}DD_{\mathrm{it}}+\gamma \prime Z_{\mathrm{it}}+\mu_{i}+\tau_{t}+\in_{\mathrm{it}}\;$$ (3)

Here, $\mu_{i}$ and $\tau_{t}$ represent individual and time fixed effects, respectively. Notably, the variable set in this model differs from that in PSM. The vector $Z_{\mathrm{it}}$ exclusively comprises time-varying covariates, including per capita household income and self-rated health. Consequently, all time-invariant variables (e.g., gender and ethnicity) were excluded from the final model, as their effects remain unidentifiable through fixed-effects estimation.

#### 2.3.4 Step 4: mechanism exploration via path analysis model

Following confirmation through PSM and FE models that the direct total effect was not significant, this investigation employed path analysis using an all-observed-variable GSEM to examine underlying complex mechanisms. This approach was applied to 2022 cross-sectional data to test the “Differentiated Social Capital Mediation Model.” GSEM facilitates clear deconstruction of the “digital divide” into multiple observable dimensions and incorporates all social capital indicators as independent observed variables. Through simultaneous estimation of multiple regression equations, it identifies both direct and indirect relationships between variables (Rabe-Hesketh and Skrondal, 2008). The selection of all-observed-variable path analysis over latent variable modeling was predicated on two considerations. First, it circumvents potential technical complications arising with latent variable models in contexts involving specific data and complex model structures, including non-convergence or non-significant factor loadings, thereby ensuring model robustness. Second, through direct examination of pathways between specific, observable indicators, findings can be translated into more explicit and actionable policy recommendations. The conceptual model is represented through the following core equations:


$${\mathrm{MH}}_{i}=c\prime {\mathrm{DD}}_{i}+{\mathrm{bSC}}_{i}+\Gamma \prime Z_{i}+\zeta_{{\mathrm{MH}}_{i}}\;$$ (4)

$${\mathrm{SC}}_{i}={\mathrm{aDD}}_{i}+{\mathrm{bSC}}_{i}+\Gamma \prime Z_{i}+\zeta_{{\mathrm{SC}}_{i}}\;$$ (5)

In this framework, Equation 4 represents the main outcome model, where mental health ( ${\mathrm{MH}}_{i}$ ) is predicted by the direct effect of the digital divide ( $c\prime {\mathrm{DD}}_{i}$ ), social capital effects ( ${\mathrm{bSC}}_{i}$ ), and a control variable vector ( $Z_{i}$ ). Equation 5 represents the mediation model, wherein social capital ( ${\mathrm{SC}}_{i}$ ) is predicted by the digital divide ( ${\mathrm{aDD}}_{i}$ ) and control variables. Complete equations detailing each observable indicator are provided in Appendix.

In accordance with best practices for GSEM applied to complex survey data, this study does not report traditional global or relative fit indices (e.g., RMSEA, CFI, AIC, BIC). This decision is based on two factors. First, our model includes non-continuous endogenous variables, necessitating the use of the GSEM, to which traditional covariance-based fit indices are not applicable. Second, our analysis must account for the complex sampling design of the CFPS (incorporating weighting, clustering, and stratification). The statistical theory for traditional fit indices assumes simple random sampling and is thus invalid in the context of complex sampling, where their application can lead to “severely biased” results (Oberski, 2014). Corroborating this methodological point, mainstream statistical software—including the Stata routine used in this study (svy: gsem)—is intentionally designed not to compute these potentially misleading indices (StataCorp, 2021). Therefore, adhering to conventions for evaluating complex GSEM models within this domain, this investigation primarily evaluates model quality through integration of three criteria: theoretical coherence, examining whether path coefficient signs align with theoretical hypotheses; local fit, emphasizing statistical significance of key path parameters; and successful model convergence status, constituting a fundamental validity prerequisite.

To precisely examine pathway significance within the model, we initially employed the lincom command to test direct path significance (a, b, and c’). Subsequently, we utilized the nlcom command, based on the Delta method, to test key indirect effect significance (a*b). The criterion for determining mediating effect significance was whether the 95% confidence interval included zero. This method demonstrates superior accuracy compared to traditional Sobel tests and maintains compatibility with this study’s complex survey design.

All analyses were performed in StataMP 17.0. The svy command or panel weights were applied throughout analyses to account for complex survey design, ensuring result accuracy and representativeness.

## 3 Results

### 3.1 Sample characteristics and the state of the digital divide

The fundamental characteristics of the study sample are delineated in Tables 1, 2. The 2022 cross-sectional data reveal that the digital divide among older adults in our sample is pronounced: a substantial 73.15% of individuals were classified as experiencing “a digital divide,” and 65.75% lacked access to a digital device. Concurrently, the sample’s social capital demonstrated a characteristic “differentiated pattern,” with mean trust in neighbors (7.04) significantly exceeding mean trust in strangers (2.42), thereby providing empirical substantiation for the subsequent differentiation between BSC and BrSC.

The panel data, conversely, demonstrate positive dynamic trajectories from 2016 to 2020. Throughout this period, the mean depression score among older adults decreased markedly by 1.15 points, while indicators including physical access, usage, and motivational access all increased substantially. Specifically, the physical access rate increased dramatically from 6.73 to 22.66%, and mean daily online time expanded from 7.02 to 28.17 min. The significant temporal variation in these core variables establishes a robust empirical foundation for employing FE modeling for causal inference.

### 3.2 Identification and operationalization of digital divide groups

To operationalize the multi-dimensional “digital divide” construct, this study employed K-means clustering. All clustering solutions satisfied statistical tests (*F*-test, *p* < 0.001), substantiating the objectivity and distinctiveness of identified groups (Tables 3, 4).

For 2022 cross-sectional data, directed toward constructing a binary treatment variable for PSM analysis, statistical tests indicated optimal CH index at *k* = 2 (847.51). The resulting composite variable (digital divide) categorized the 4,102 older adults into a “without digital divide” group (control group, *N* = 1,101, 26.85%) and a “with digital divide” group (treatment group, *N* = 3,001, 73.15%) (Table 1).

For 2016–2020 panel data, the CH index likewise demonstrated optimality at *k* = 2 (8950.77), while the Elbow Method indicated *k* = 3 and *k* = 4 as equally optimal. Consequently, we retained all three clustering solutions (*k* = 2, 3, and 4) for subsequent robustness assessments. Notably, the proportion of the “with digital divide” group in 2022 was substantially lower than in preceding years (84.57% in panel data for *k* = 2), reflecting the digital divide’s dynamic evolution over time and underscoring the necessity of analyzing the two datasets independently (Tables 2, 3).

### 3.3 The impact of the digital divide on mental health: robust null-effect evidence from PSM

This investigation utilized 1:4 nearest neighbor matching as the primary model. Balance test results demonstrated that the matching procedure significantly enhanced sample balance (Table 5). Following matching, the mean standardized bias decreased from 20.7 to 3.4%, and Rubin’s B statistic fell within the optimal range (18.9 < 25). Biases for the preponderance of key covariates remained below 10%, and their t-test results ceased to achieve significance (Table 6). These findings demonstrate that PSM successfully eliminated systematic differences in observable variables.

**Table 5:** Covariate balance test results before and after 1-to-4 nearest neighbor matching.

Variable	Sample	Mean (treated)	Mean (control)	% Std. bias	*p*-value (*t*-test)
Control variables					
Age (years)	Unmatched	69.09	66.141	52.4	<0.001
	Matched	69.001	68.787	3.8	0.16
Gender	Unmatched	0.50672	0.55756	−10.2	0.003
	Matched	0.50757	0.52884	−4.3	0.094
Ethnicity	Unmatched	1.0614	1.0447	7.5	0.035
	Matched	1.0619	1.0491	5.7	0.027
Marital status	Unmatched	0.81926	0.87543	−15.7	<0.001
	Matched	0.82146	0.80664	4.1	0.133
Number of children	Unmatched	0.98401	1.0619	−11	0.001
	Matched	0.98421	0.97374	1.5	0.553
Education	Unmatched	2.0765	2.817	−68.7	<0.001
	Matched	2.0822	2.1467	−6	0.017
LnPCHI	Unmatched	9.5371	10.106	−48.7	<0.001
	Matched	9.5476	9.5979	−4.3	0.126
Residence	Unmatched	0.44754	0.62457	−36.1	<0.001
	Matched	0.44989	0.47035	−4.2	0.106
Self-rated social status	Unmatched	3.4514	3.4948	−4	0.252
	Matched	3.4541	3.5149	−5.6	0.032
Life satisfaction	Unmatched	4.2006	4.2878	−10.4	0.004
	Matched	4.2059	4.2124	−0.8	0.774
Confidence in future	Unmatched	4.1216	4.3393	−24.1	<0.001
	Matched	4.1247	4.1926	−7.5	0.005
Region	Unmatched	1.8052	1.7749	3.8	0.27
	Matched	1.807	1.7857	2.7	0.298
Basic social medical insurance	Unmatched	1.0345	1.189	−30.1	<0.001
	Matched	1.0354	1.0393	−0.8	0.748
Self-rated health	Unmatched	3.4069	3.1641	19.9	<0.001
	Matched	3.4009	3.3544	3.8	0.154
Chronic disease	Unmatched	0.32502	0.30928	3.4	0.326
	Matched	0.32549	0.296	6.3	0.012
Exercise frequency	Unmatched	0.61004	0.89605	−31.5	<0.001
	Matched	0.6136	0.6194	−0.6	0.796
Social capital					
Neighbor trust	Unmatched	0.68106	0.74742	−14.7	<0.001
	Matched	0.68321	0.68329	−0.001	0.995
LnFinancial transfers	Unmatched	2.72	2.4957	5.8	0.091
	Matched	2.7155	2.7478	−0.8	0.743
Child–parent relationship	Unmatched	4.2365	4.3958	−17.3	<0.001
	Matched	4.2414	4.2655	−2.6	0.316
Contact frequency	Unmatched	2.4292	2.363	4.7	0.175
	Matched	2.4305	2.3563	5.2	0.039
Communication frequency	Unmatched	2.7373	2.3774	30.7	<0.001
	Matched	2.729	2.7068	1.9	0.464
Trust in strangers	Unmatched	2.3356	2.4923	−6.5	0.062
	Matched	2.3181	2.3344	−0.7	0.798
Interpersonal relationships	Unmatched	0.64459	0.7311	−18.7	<0.001
	Matched	0.64679	0.6277	4.1	0.118

**Table 6:** Propensity score test to assess quality of matching.

Sample	Ps R2	LR chi2	*p* > chi2	Mean bias	Med bias	Rubin’s *B*	Rubin’s *R*
Unmatched	0.153	765.53	<0.001	20.7	15.7	100.7*	1.21
Matched	0.006	55.21	<0.001	3.4	3.8	18.9	1.37

^*^if *B* > 25%, *R* outside [0.5, 2]; % Std. bias refers to the percentage of standardized bias.

ATT estimation revealed that, after controlling for observable confounding variables, the direct causal effect of the digital divide on older adults’ mental health was not significant. As presented in Table 7, the ATT estimate from the 1:4 nearest neighbor matching model approached zero (0.02, *t* = 0.10). This “null effect” conclusion remained robust following application of various matching algorithms (1,1 nearest neighbor, caliper, kernel) and utilization of a 500-repetition bootstrap procedure.

**Table 7:** Estimation of average treatment effect on the treated under different matching methods.

Matching method	ATT	Std. err.	*t*/*z*-statistic	*p*-value
1:1 nearest neighbor	−0.02	0.28	−0.05	>0.1
*Bootstrap SE*	0.19	0.35	0.55	0.58
1:4 nearest neighbor	0.02	0.22	0.10	>0.1
*Bootstrap SE*	0.00	0.33	0.00	1.00
Caliper matching (0.25 SD)	−0.02	0.28	−0.05	>0.1
*Bootstrap SE*	−0.54	0.35	−1.55	0.12
Kernel matching	0.15	0.18	0.85	>0.1
*Bootstrap SE*	−0.02	0.26	−0.06	0.95

Rows in italics represent the standard errors and corresponding *z*/*p*-values obtained using the bootstrap method (500 repetitions).

To further substantiate conclusion reliability, we implemented a series of robustness and sensitivity assessments. Whether employing alternative treatment variable definitions (re-analyzing exclusively among internet users, see Supplementary Table S1), examining potential hidden bias through Rosenbaum bounds sensitivity analysis (see Supplementary Table S2), or conducting heterogeneity analysis by gender and residence (see Table 8 and Supplementary Table S3), all tests consistently indicated the same conclusion: insufficient evidence exists to support a general and robust direct causal effect of the digital divide on older adults’ mental health.

**Table 8:** Heterogeneity analysis of the ATT by gender and residence.

Group	Subgroup	Matching method	ATT	Std. err.	*t*/*z*-statistic	*p*-value
Gender	Female	1:1 Nearest neighbor	−0.28	0.39	−0.72	> 0.1
		*Bootstrap SE*	−0.74	0.50	−1.46	0.14
		Kernel matching	−0.12	0.28	−0.43	>0.1
		*Bootstrap SE*	0.23	0.43	0.55	0.58
	Male	1:1 Nearest neighbor	0.23	0.34	0.69	>0.1
		*Bootstrap SE*	0.17	0.45	0.38	0.71
		Kernel matching	0.36	0.23	1.58	>0.1
		*Bootstrap SE*	0.28	0.35	0.80	0.43
Residence	Rural	1:1 Nearest neighbor	0.11	0.39	0.27	>0.1
		*Bootstrap SE*	−0.49	0.52	−0.93	0.35
		Kernel matching	0.25	0.29	0.85	>0.1
		*Bootstrap SE*	−0.25	0.40	−0.63	0.53
	Urban	1:1 Nearest neighbor	−0.16	0.33	−0.50	>0.1
		*Bootstrap SE*	−0.02	0.46	−0.04	0.97
		Kernel matching	0.02	0.23	0.07	>0.1
		*Bootstrap SE*	0.07	0.34	0.22	0.83

Rows in italics represent the standard errors and corresponding z/p-values obtained using the bootstrap method (500 repetitions).

### 3.4 The impact of the digital divide on mental health: re-validation after controlling for time-invariant heterogeneity

To address bias from time-invariant, unobservable individual heterogeneity, this study employed FE modeling for longitudinal causal inference, a selection strongly substantiated by the Hausman test result (*p* < 0.0001). As demonstrated in Table 9, the digital divide coefficient remained non-significant (*p* > 0.05) across all model specifications, irrespective of clustering definition (*k* = 2, 3, or 4) utilized as the core explanatory variable or whether region fixed effects were additionally controlled. This finding demonstrates strong consistency with PSM analysis, indicating that the digital divide lacks statistically significant direct effects on older adults’ mental health. The superficial association observed between these variables is therefore likely attributable to the combined influence of observable socioeconomic characteristics and unobservable, time-invariant individual attributes.

**Table 9:** Panel fixed-effects models of the impact of the digital divide on mental health.

Variable	Two-way FE models			Three-way FE models		
	Digital divide (*k* = 4)	Digital divide (*k* = 3)	Digital divide (*k* = 2)	Digital divide (*k* = 4)	Digital divide (*k* = 3)	Digital divide (*k* = 2)
Digital divide (*k* = 4) (reference group: without digital divide)						
Medium digital divide	0.407			0.407		
	(0.222)			(0.227)		
High digital divide	0.334			0.334		
	(0.212)			(0.218)		
Very high digital divide	0.370			0.370		
	(0.276)			(0.275)		
Digital divide (*k* = 3) (reference group: without digital divide)						
Medium digital divide		−0.034			−0.034	
		(0.186)			(0.193)	
High digital divide		0.092			0.092	
		(0.225)			(0.228)	
Digital divide (*k* = 2) (reference group: without digital divide)						
With digital divide			0.054			0.054
			(0.184)			(0.187)
Neighbor trust (reference group: low)						
Middle	−0.014	−0.020	−0.017	−0.014	−0.020	−0.017
	(0.153)	(0.153)	(0.153)	(0.158)	(0.158)	(0.158)
high	−0.108	−0.102	−0.106	−0.108	−0.102	−0.106
	(0.133)	(0.133)	(0.133)	(0.146)	(0.146)	(0.146)
LnFinancial transfers	0.027	0.027	0.027	0.027	0.027	0.027
	(0.015)	(0.015)	(0.015)	(0.015)	(0.015)	(0.015)
Child–parent relationship	−0.401*	−0.401*	−0.403*	−0.401*	−0.401*	−0.403*
	(0.101)	(0.101)	(0.101)	(0.102)	(0.102)	(0.102)
Contact frequency	0.001	−0.000	−0.000	0.001	−0.000	−0.000
	(0.049)	(0.048)	(0.048)	(0.049)	(0.049)	(0.049)
Communication frequency	0.018	0.016	0.017	0.018	0.016	0.017
	(0.045)	(0.046)	(0.046)	(0.046)	(0.046)	(0.046)
Trust in strangers (reference group: low)						
High	−0.083	−0.078	−0.082	−0.083	−0.078	−0.082
	(0.148)	(0.148)	(0.148)	(0.150)	(0.150)	(0.149)
Age (Years)	0.330	0.334	0.334	0.330	0.334	0.334
	(0.220)	(0.219)	(0.219)	(0.214)	(0.214)	(0.214)
Marital status (reference group: divorced/widowed/single/cohabiting)						
Married	−0.864	−0.852	−0.855	−0.864	−0.852	−0.855
	(0.490)	(0.493)	(0.493)	(0.539)	(0.541)	(0.542)
Number of children (reference group: 0)						
1	0.383	0.383	0.379	0.383	0.383	0.379
	(0.440)	(0.442)	(0.441)	(0.431)	(0.433)	(0.432)
2 or more	0.092	0.104	0.097	0.092	0.104	0.097
	(0.550)	(0.550)	(0.551)	(0.549)	(0.550)	(0.551)
Education (reference group: illiterate/semi-literate)						
Primary school	1.047	1.034	1.046	1.047	1.034	1.046
	(0.564)	(0.560)	(0.564)	(0.578)	(0.575)	(0.579)
Lower secondary school	3.044	3.116	3.092	3.044	3.116	3.092
	(1.772)	(1.803)	(1.807)	(1.713)	(1.757)	(1.757)
Upper secondary education and above	−1.364	−1.328	−1.325	−1.364	−1.328	−1.325
	(1.327)	(1.308)	(1.307)	(1.258)	(1.242)	(1.242)
LnPCHI	−0.125	−0.127	−0.127	−0.125	−0.127	−0.127
	(0.074)	(0.074)	(0.074)	(0.073)	(0.073)	(0.073)
Residence (reference group: rural)						
Urban	−0.591	−0.598	−0.604	−0.591	−0.598	−0.604
	(0.396)	(0.394)	(0.395)	(0.393)	(0.390)	(0.392)
Self-rated social status (reference group: very low)						
Low	0.209	0.194	0.195	0.209	0.194	0.195
	(0.239)	(0.239)	(0.239)	(0.245)	(0.245)	(0.245)
Average	−0.226	−0.228	−0.232	−0.226	−0.228	−0.232
	(0.223)	(0.223)	(0.222)	(0.231)	(0.231)	(0.231)
High	−0.239	−0.235	−0.241	−0.239	−0.235	−0.241
	(0.264)	(0.265)	(0.263)	(0.266)	(0.266)	(0.265)
Very high	−0.268	−0.253	−0.262	−0.268	−0.253	−0.262
	(0.274)	(0.276)	(0.274)	(0.277)	(0.279)	(0.277)
Wellbeing index	−0.502*	−0.496*	−0.501*	−0.502*	−0.496*	−0.501*
	(0.079)	(0.079)	(0.079)	(0.081)	(0.080)	(0.081)
Region (reference group: eastern)						
Central	−1.512	−1.400	−1.441			
	(0.856)	(0.863)	(0.866)			
Western	−1.287	−1.334	−1.311			
	(1.172)	(1.175)	(1.175)			
Basic social medical insurance (reference group: not enrolled)						
URRBMI	−0.491*	−0.494*	−0.493*	−0.491*	−0.494*	−0.493*
	(0.207)	(0.207)	(0.207)	(0.211)	(0.211)	(0.211)
UEBMI/government-funded medical care	0.131	0.130	0.136	0.131	0.130	0.136
	(0.266)	(0.267)	(0.267)	(0.286)	(0.286)	(0.286)
Self-rated health (reference group: extremely healthy)						
Very healthy	0.109	0.123	0.122	0.109	0.123	0.122
	(0.234)	(0.235)	(0.235)	(0.235)	(0.235)	(0.235)
Relatively healthy	0.611*	0.633*	0.629*	0.611*	0.633*	0.629*
	(0.215)	(0.215)	(0.216)	(0.214)	(0.214)	(0.215)
Average	0.936*	0.955*	0.955*	0.936*	0.955*	0.955*
	(0.238)	(0.238)	(0.239)	(0.238)	(0.238)	(0.238)
Unhealthy	1.890*	1.908*	1.908*	1.890*	1.908*	1.908*
	(0.252)	(0.252)	(0.252)	(0.252)	(0.252)	(0.253)
Chronic disease (reference group: without)						
With	0.407*	0.411*	0.410*	0.407*	0.411*	0.410*
	(0.140)	(0.140)	(0.141)	(0.144)	(0.144)	(0.145)
Exercise frequency (reference group: never/less than once a week)						
1–6 times a week	0.271	0.256	0.257	0.271	0.256	0.257
	(0.179)	(0.179)	(0.178)	(0.187)	(0.187)	(0.187)
Daily	−0.101	−0.104	−0.104	−0.101	−0.104	−0.104
	(0.144)	(0.144)	(0.144)	(0.148)	(0.148)	(0.148)
Individual FE	Yes	Yes	Yes	Yes	Yes	Yes
Region FE	No	No	No	Yes	Yes	Yes
Year FE	Yes	Yes	Yes	Yes	Yes	Yes
_cons	−12.587	−12.521	−12.534	−13.857	−13.778	−13.800
	(14.897)	(14.852)	(14.867)	(14.798)	(14.769)	(14.782)
*N*	18,553	18,553	18,553	14,963	14,963	14,963
Within *R*^2^	0.0772	0.0768	0.0766	0.0556	0.0552	0.0550

*, **, and *** denote significance at the 5, 1%, and 1‰ levels, respectively. SE, standard error; PCHI, *per capita* household income; CNY, Chinese yuan; UEBMI, urban employee basic medical insurance. URRBMI, urban and rural basic resident medical insurance.

### 3.5 Decomposing the null effect: a path analysis of the suppression effect of the digital divide via social capital

**Figure 2:**
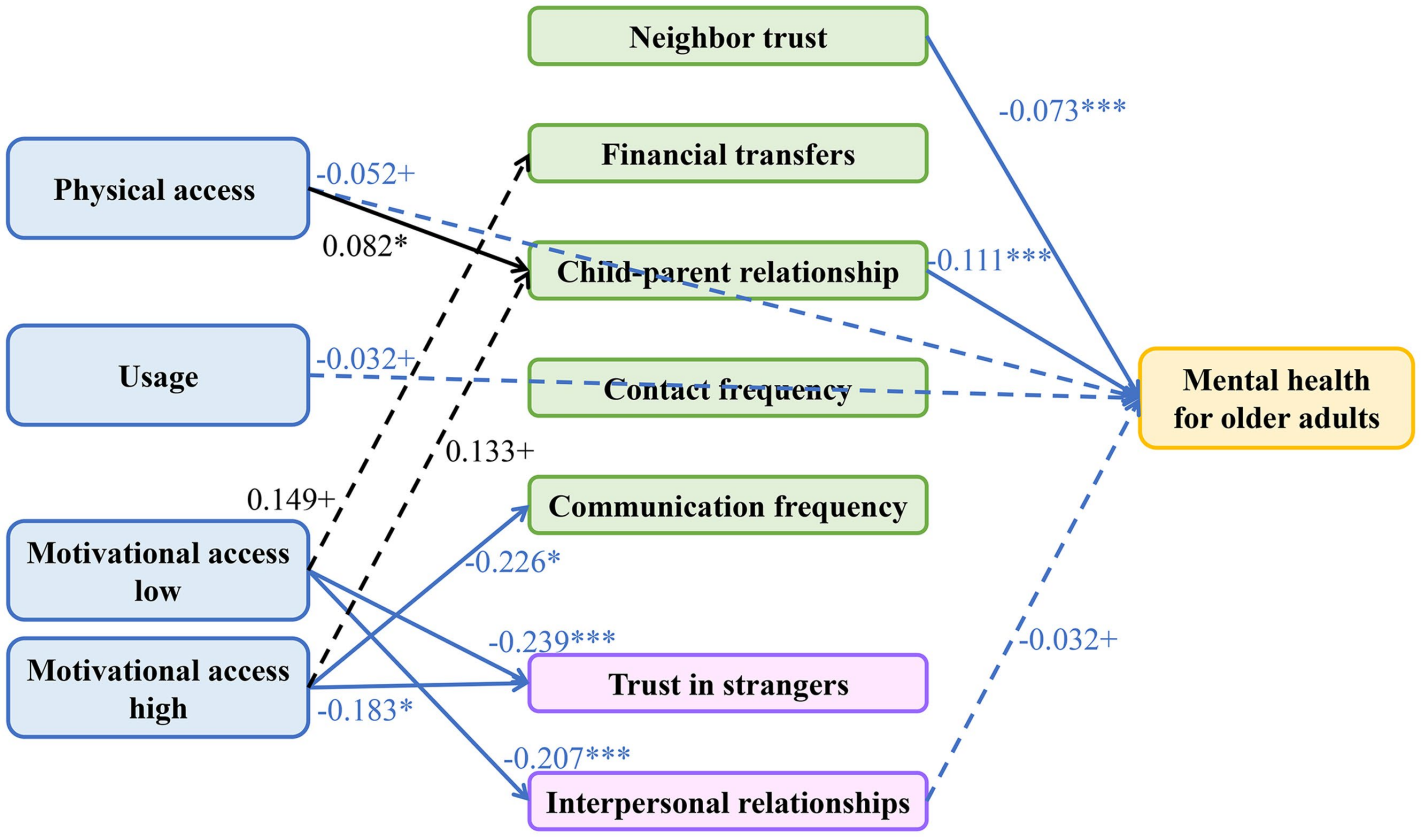
Path analysis of the significant pathways linking the digital divide, social capital, and mental health. Values on the paths are standardized coefficients. The model accounted for the correlations of some error terms. Significance levels: ***p < 0.001, **p < 0.01, *p < 0.05, ^+^*p* < 0.1. Line style indicates significance: solid lines represent paths significant at *p* < 0.05, while dashed lines represent paths marginally significant at *p* < 0.1. Arrow and coefficient color indicates the direction of the effect: black arrows represent positive effects, and blue arrows represent negative effects.

Following PSM and FE models revealing no significant total effect of the digital divide on older adults’ mental health, we employed path analysis using an all-observed-variable GSEM to elucidate complex underlying mechanisms. Model results (see Tables 10, 11 and Figure 2) unveiled a classic inconsistent mediation pattern, namely a suppression effect. This implies that the digital divide has both direct and indirect pathways to mental health that operate in opposite directions, counteracting each other and thereby suppressing the total effect, which elucidates the non-significant findings from broader causal analyses. We deconstruct this mechanism systematically below.

**Table 10:** Direct effect estimates from the GSEM predicting depressive symptoms.

Path	*B*	*β*	*t*	*p*-value	95% CI
Physical access → mental health	−0.491+	−0.052	−1.94	0.055	(−0.991, 0.010)
Usage → mental health	−0.145+	−0.032	−1.73	0.085	(−0.311, 0.020)
Motivational access_low → mental health	−0.073 ns	−0.016	−0.29	0.774	(−0.578, 0.431)
Motivational access_high → mental health	0.192 ns	0.043	0.85	0.397	(−0.255, 0.640)
Neighbor trust → mental health	−0.715***	−0.073	−4.85	<0.001	(−1.005, −0.424)
Child–parent relationship → mental health	−0.502***	−0.111	−5.39	<0.001	(−0.686, −0.318)
Financial transfers → mental health	0.050 ns	0.011	0.64	0.526	(−0.105, 0.205)
Contact frequency → mental health	0.111 ns	0.025	1.17	0.243	(−0.076, 0.299)
Communication frequency → mental health	0.030 ns	0.007	0.35	0.73	(−0.143, 0.203)
Trust in strangers → mental health	0.003 ns	0.001	0.07	0.941	(−0.067, 0.072)
Interpersonal relationships → mental health	−0.307+	−0.032	−1.69	0.093	(−0.665, 0.052)
Physical access → neighbor trust	−0.054 ns	−0.056	−1.65	0.101	(−0.119, 0.011)
Physical access → child–parent relationship	0.173*	0.082	2.01	0.046	(0.003, 0.344)
Physical access → financial transfers	0.035 ns	0.017	0.48	0.631	(−0.108, 0.178)
Physical access → contact frequency	−0.021 ns	−0.01	−0.33	0.743	(−0.145, 0.104)
Physical access → communication frequency	0.012 ns	0.006	0.15	0.877	(−0.142, 0.166)
Physical access → trust in strangers	0.208 ns	0.04	1.4	0.165	(−0.086, 0.502)
Physical access → Interpersonal relationships	0.020 ns	0.021	0.66	0.51	(−0.041, 0.081)
Usage → neighbor trust	0.010 ns	0.022	0.76	0.446	(−0.016, 0.036)
Usage → child–parent relationship	0.006 ns	0.006	0.22	0.824	(−0.045, 0.057)
Usage → financial transfers	−0.026 ns	−0.026	−0.74	0.461	(−0.095, 0.043)
Usage → contact frequency	−0.012 ns	−0.012	−0.49	0.624	(−0.062, 0.037)
Usage → communication frequency	−0.018 ns	−0.018	−0.71	0.476	(−0.067, 0.031)
Usage → trust in strangers	0.043 ns	0.017	0.65	0.518	(−0.087, 0.172)
Usage → interpersonal relationships	−0.016 ns	−0.034	−1.24	0.217	(−0.042, 0.010)
Motivational access_low → neighbor trust	−0.029 ns	−0.063	−1.13	0.261	(−0.079, 0.022)
Motivational access_low → child–parent relationship	0.027 ns	0.027	0.32	0.752	(−0.142, 0.196)
Motivational access_low → financial transfers	0.149+	0.149	1.78	0.077	(−0.016, 0.313)
Motivational access_low → contact frequency	−0.021 ns	−0.021	−0.32	0.749	(−0.151, 0.109)
Motivational access_low → communication frequency	−0.064 ns	−0.064	−0.74	0.46	(−0.234, 0.106)
Motivational access_low → trust in strangers	−0.583***	−0.239	−3.46	0.001	(−0.915, −0.250)
Motivational access_low → interpersonal relationships	−0.098***	−0.207	−3.75	<0.001	(−0.149, −0.046)
Motivational access_high → neighbor trust	0.043 ns	0.093	1.27	0.207	(−0.024, 0.109)
Motivational access_high → child–parent relationship	0.04 ns	0.04	0.49	0.627	(−0.121, 0.201)
Motivational access_high → financial transfers	0.133+	0.133	1.93	0.055	(−0.003, 0.269)
Motivational access_high → contact frequency	0.041 ns	0.041	0.54	0.59	(−0.108, 0.190)
Motivational access_high → Communication frequency	−0.226*	−0.226	−2.54	0.012	(−0.402, −0.050)
Motivational access_high → Trust in strangers	−0.446*	−0.183	−2.55	0.012	(−0.791, −0.101)
Motivational access_high → Interpersonal relationships	0.041 ns	0.088	1.32	0.189	(−0.021, 0.103)

*B*, unstandardized coefficient; *β*, standardized coefficient. Standard errors are in parentheses. All control variables were included in the model but are not shown for brevity. ns *p* > 0.1,^+^*p* < 0.1, *p < 0.05, **p < 0.01, ***p < 0.001.

**Table 11:** Indirect and total effects of the digital divide on depressive symptoms.

Variable	Effect type	*B*	*β*	*t/z*	*p*-value	95% CI	Proportion mediated (%)
Physical access	Indirect effects						
	→Neighbor trust → mental health	0.039 ns	0.004	1.57	0.116	(−0.010, 0.087)	0.00%
	→Child–parent relationship → mental health	−0.087+	−0.009	−1.8	0.072	(−0.182, 0.008)	0.00%
	→Financial transfers → mental health	0.002 ns	0.000	0.38	0.701	(−0.007, 0.011)	0.00%
	→Contact frequency → mental health	−0.002 ns	<−0.001	−0.31	0.758	(−0.017, 0.012)	0.00%
	→Communication frequency → mental health	<0.001 ns	<0.001	0.15	0.878	(−0.004, 0.005)	0.00%
	→Trust in strangers → mental health	0.001 ns	<0.001	0.07	0.941	(−0.014, 0.015)	0.00%
	→Interpersonal relationships → Mental health	−0.006 ns	−0.001	−0.61	0.539	(−0.026, 0.014)	0.00%
	Total effects	−0.545*	−0.057	−2.09	0.037	(−1.057, −0.033)	−10.53%
Usage	Indirect effects						
	→Neighbor trust → mental health	−0.007 ns	−0.002	−0.74	0.457	(−0.026, 0.012)	0.00%
	→Child–parent relationship → mental health	−0.003 ns	−0.001	−0.22	0.824	(−0.028, 0.023)	0.00%
	→Financial transfers → mental health	−0.001 ns	<−0.001	−0.45	0.651	(−0.007, 0.004)	0.00%
	→Contact frequency → mental health	−0.001 ns	<−0.001	−0.44	0.658	(−0.007, 0.005)	0.00%
	→Communication frequency → mental health	−0.001 ns	<−0.001	−0.34	0.736	(−0.004, 0.003)	0.00%
	→Trust in strangers → mental health	<0.001 ns	<0.001	0.07	0.941	(−0.003, 0.003)	0.00%
	→Interpersonal relationships → mental health	0.005 ns	0.001	0.99	0.32	(−0.005, 0.015)	0.00%
	Total effects	−0.154+	−0.034	−1.76	0.078	(−0.324, 0.017)	23.53%
Motivational access_low	Indirect effects						
	→Neighbor trust → mental health	0.021 ns	0.005	1.07	0.284	(−0.017, 0.058)	0.00%
	→Child–parent relationship → mental health	−0.014 ns	−0.003	−0.32	0.752	(−0.098, 0.071)	0.00%
	→Financial transfers → mental health	0.007 ns	0.002	0.56	0.574	(−0.018, 0.033)	0.00%
	→Contact frequency → mental health	−0.002 ns	−0.001	−0.31	0.753	(−0.017, 0.012)	0.00%
	→Communication frequency → mental health	−0.002 ns	<−0.001	−0.33	0.743	(−0.013, 0.010)	0.00%
	→Trust in strangers → mental health	−0.002 ns	<−0.001	−0.07	0.941	(−0.042, 0.039)	0.00%
	→Interpersonal relationships → Mental health	0.03 ns	0.007	1.56	0.118	(−0.008, 0.067)	0.00%
	Total effects	−0.035 ns	−0.008	−0.14	0.892	(−0.540, 0.470)	−112.50%
Motivational access_high	Indirect effects						
	→Neighbor trust → mental health	−0.03 ns	−0.007	−1.24	0.213	(−0.078, 0.017)	0.00%
	→Child–parent relationship → mental health	−0.02 ns	−0.004	−0.49	0.621	(−0.099, 0.059)	0.00%
	→Financial transfers → mental health	0.007 ns	0.001	0.56	0.574	(−0.016, 0.030)	0.00%
	→Contact frequency → mental health	0.005 ns	0.001	0.47	0.639	(−0.014, 0.024)	0.00%
	→Communication frequency → mental health	−0.007 ns	−0.002	−0.35	0.726	(−0.045, 0.031)	0.00%
	→Trust in strangers → mental health	−0.001 ns	<−0.001	−0.07	0.941	(−0.032, 0.029)	0.00%
	→Interpersonal relationships → mental health	−0.013 ns	−0.003	−1.07	0.286	(−0.036, 0.011)	0.00%
	Total effects	0.132 ns	0.029	0.56	0.578	(−0.334, 0.599)	−3.45%

*B*, unstandardized coefficient; *β*, standardized coefficient. Standard errors are in parentheses. All control variables were included in the model but are not shown for brevity. ns *p* > 0.1,^+^*p* < 0.1, *p < 0.05, **p < 0.01, ***p < 0.001.

First, we identified a beneficial direct pathway (path *c*’). Results showed that after controlling for all social capital mediators, digital participation itself had a direct protective effect on mental health. Specifically, physical access and usage both demonstrated marginally significant, negative direct effects on depressive symptoms (for physical access: *β* = −0.052, *p* < 0.1; for usage: *β* = −0.032, *p* < 0.1) (Table 10). This indicates that internet engagement itself, possibly through channels including information access or entertainment, has a direct and beneficial impact on older adults’ mental health, constituting the “beneficial direct pathway” of the suppression model.

Second, we identified a harmful indirect pathway (path a*b), whose formation was validated through two steps. Step one, results for path *a* (digital divide → social capital) revealed that higher-order “motivational access” exerted pronounced erosive effect on BrSC. Compared to non-users, low-perception users demonstrated significantly lower scores on trust in strangers (*β* = −0.239, *p* = 0.001) and interpersonal relationships (*β* = −0.207, *p* < 0.001). This constitutes the “harmful originating path” of the indirect effect. Step two, results for path *b* (social capital → mental health) indicated that multiple social capital forms exerted significant protective effects. BSC indicators, encompassing neighbor trust (*β* = −0.073, *p* < 0.001) and child–parent relationship (*β* = −0.111, *p* < 0.001), alongside the BrSC indicator interpersonal relationships (*β* = −0.032, *p* = 0.093), all significantly reduced older adults’ depression levels. This constitutes the “beneficial transmission path” of the indirect effect. Integrating these two steps, a complete harmful indirect pathway was substantiated: “motivational access” erodes interpersonal relationships (negative path a), and enhanced interpersonal relationships subsequently reduce depression (negative path *b*). The product of these paths (negative × negative = positive) constitutes a harmful pathway with positive effects, signifying that motivational access ultimately increases depression through social capital degradation.

Synthesizing these findings, the full picture of the “suppression effect” emerged: beneficial direct pathway and harmful indirect pathways coexist. These opposing forces compete with and neutralize each other, ultimately producing the ostensibly null “zero total effect” observed in PSM and FE models. This finding additionally provides reasonable interpretation for apparently anomalous statistical results in Table 11: statistical non-significance of specific indirect effects, total indirect effects, and presence of negative or extreme indirect effect ratios (e.g., −112.50%) do not constitute evidence of “no mediation.” Rather, they represent statistical manifestations of complex dynamics wherein multiple, opposing pathways intertwine and suppress one another.

## 4 Discussion

Employing a multi-method strategy, this investigation systematically analyzed the complex association between the digital divide, social capital, and mental health among older adults in China. The principal finding reveals a statistical suppression effect (Jacob Cohen et al., 2003): direct psychological benefits of digital participation are substantially counteracted by indirect social costs incurred through social capital erosion. This finding not only provides a novel causal framework for understanding “active aging” in the digital era but also furnishes empirical substantiation for the hypotheses advanced in this investigation.

### 4.1 Re-examining the “null total effect”: verification of H1

The primary finding, derived from both PSM and FE models, yields a highly robust conclusion: after rigorously controlling for self-selection bias and individual heterogeneity, the direct total effect of the digital divide on older adults’ mental health is not significant. This “robust null effect” finding strongly substantiates H1 and positions our investigation at the center of a fundamental debate within the field.

This result appears to contradict conclusions from several large-scale investigations that identified significant associations between digital divide and elevated depressive symptoms (Barreda Gutiérrez et al., 2024). Conversely, our finding aligns with substantial literature reporting mixed, inconsistent, or null effects (Rosell et al., 2023; Nimrod, 2020). Indeed, systematic reviews have indicated that assertions of simple, direct benefits of digital technology for mental health have frequently been overstated due to methodological limitations in preceding research (Liu et al., 2025).

Our investigation’s multi-method design provides evidence to elucidate this contradiction. Simple associations observed in numerous early studies likely originated from inadequately addressed self-selection bias (Liu et al., 2025); for instance, healthier, wealthier, more educated, and more socially active older adults naturally demonstrate greater propensity to utilize the internet while possessing superior mental health (Solar and Irwin, 2010; Friemel, 2016). The PSM methodology we employed mitigated this bias through matching individuals on extensive observable variables, while the FE model advanced further by analyzing within-individual changes, thereby controlling for time-invariant unobserved heterogeneity (e.g., inherent personality traits or cognitive abilities). Consequently, the direct association documented in previous literature dissipated in our analysis. This represents not a failure to identify an association, but rather successful demonstration that robust direct causal pathways between these constructs do not exist after accounting for confounding effects. The substantive implication of this finding suggests that policies anticipating direct and universal mental health improvements among older adults merely through bridging “physical access” may be excessively optimistic; the genuine impact of the digital divide must be comprehended through more complex, indirect mechanisms.

### 4.2 The tale of two capitals: how the digital divide differentially shapes social resources

Following establishment of the non-significant total effect, this study’s core objective shifts to elucidating underlying mechanisms. Path analysis results clearly delineate the differentiated impact of the digital divide on distinct social capital types, providing robust support for H2.

First, the investigation revealed that physical access significantly strengthened BSC, positively predicting child–parent relationship (*β* = 0.082, *p* < 0.05). This finding substantiates expectations of H2a and furnishes empirical evidence for Socioemotional Selectivity Theory (SST) in the digital age (English and Carstensen, 2017). This theory postulates that older adults prioritize emotionally meaningful relationships, particularly familial bonds (Simons et al., 2023). Our results indicate that physical access serves as an efficient instrument for older adults to achieve this fundamental social objective. Through facilitating contact maintenance with non-co-resident relatives (Hwang et al., 2022; Bardach et al., 2021), it effectively strengthens their BSC, findings consonant with prior research (Simons et al., 2023; Song et al., 2021).

In sharp contrast, a more innovative finding strongly supports H2b: higher-order “motivational access” exerts pronounced erosive effect on BrSC. Results show that users with lower perceived importance of the internet also reported significantly lower trust in strangers and poorer self-rated interpersonal relationships. We theorize this originates from a key psychosocial mechanism: deficient digital literacy amplifies online risk perception (Aleti et al., 2025), and this risk perception generalizes from digital domains to pervasive social distrust (Sabatini and Sarracino, 2019), forming psychological barriers to establishing weak ties (BrSC’s essence) and ultimately precipitating novel forms of social exclusion. In short, the harm of motivational access extends beyond the technological realm; through reshaping older adults’ fundamental societal risk perceptions, it cultivates “defensive psychology” antithetical to open and diverse social integration.

A more thought-provoking finding reveals that this social capital erosion is more pervasive than anticipated, affecting even “high-perception users.” Results indicate that elevated motivational access also significantly reduced older adults’ trust in strangers (*β* = −0.183, *p* < 0.05) and communication frequency with children (*β* = −0.226, *p* < 0.05). Notably, however, this erosion of specific social capital forms did not ultimately manifest as significant mental health deterioration, as their pathways to depressive symptoms (path *b*) lacked statistical significance. This suggests two profound mechanisms: first, maintaining traditional social connections in the digital age may constitute a universal challenge for all older adults; second, among various social capital forms, “interpersonal relationships” may function as a special “last line of defense,” as once this more comprehensive social perception experiences erosion, negative consequences transmit immediately and significantly to individual mental health.

### 4.3 The architecture of protection: primacy of BSC and the ambivalence of intergenerational support

This study’s examination of differential social capitals’ direct protective effects provides strong support for H3a: BSC functions as the core protective layer for older adults’ mental health. Path analysis results clearly demonstrate that both trust in neighbors and emotional quality of child–parent relationships powerfully and significantly reduce depressive symptoms. This indicates that for China’s older adult population, social support originating from close-knit communities and core family units constitutes the primary and most reliable resource for mitigating psychological risks (Tengku Mohd et al., 2019).

Concurrently, results provide partial evidence supporting H3b, as BrSC, measured through interpersonal relationships, demonstrated marginally significant protective effect. However, a particularly noteworthy and anomalous finding emerged: financial support received from non-co-resident relatives exhibited no significant mental health effects. This “null effect” challenges simple economic models—which postulate that increased resources reduce stress and enhance wellbeing—yet aligns closely with intergenerational ambivalence theory. This theory proposes that financial support represents a “double-edged sword” for older adults: while materially beneficial and symbolizing filial piety, it simultaneously signifies status reversal, independence loss, and confirmation of constituting a “burden” to children, thereby inducing negative emotions including guilt or diminished self-worth (Pillemer and Lüscher, 2003). Therefore, the non-significant coefficient for financial transfers likely represents not an “absence of effect,” but rather the statistical outcome of these opposing psychological forces neutralizing each other. This finding profoundly reveals that for older adults’ mental health, relationship emotional quality far exceeds material exchange importance.

### 4.4 Revealing complexity: confirmation of H4 and elucidation of the suppression effect

Synthesizing all preceding findings, this investigation ultimately confirms H4 comprehensively and reveals its most significant theoretical contribution: beneath the ostensibly null total effect lies a suppression effect, constituted by conflicting direct and indirect effects.

Our path analysis clearly reveals two opposing forces. On one hand, a beneficial direct pathway (path *c*’) exists: even excluding social capital’s mediating role, internet engagement itself can directly, albeit marginally, reduce older adults’ depression levels. On the other hand, a harmful indirect pathway (path a*b) operates: higher-order motivational access erodes BrSC (negative path *a*), and BrSC subsequently protects mental health (negative path *b*). The product of these paths constitutes a harmful pathway with a positive effect, signifying that motivational access indirectly increases depression through social capital degradation. Competition and mutual cancellation between these opposing forces ultimately produce the ostensibly null “zero total effect” observed in PSM and FE models. This phenomenon is recognized statistically as a suppression effect, or “inconsistent mediation” (Jacob Cohen et al., 2003).

This finding provides crucial empirical evidence resolving the academic debate introduced earlier. It suggests that the future research question should no longer address “whether the digital divide affects mental health,” but rather “how positive, direct technological benefits and negative, indirect social risks achieve complex balance within different individuals.” Therefore, this investigation’s core theoretical contribution lies not in simply interpreting the “null total effect” as impact absence. Instead, through rigorous empirical modeling, it clearly reveals underlying suppression effects, providing a more comprehensive and precise causal framework for understanding the digital divide’s genuine impact.

### 4.5 The role of the COVID-19 pandemic as a confounding context

A significant contextual factor for this study is the COVID-19 pandemic, the onset of which overlaps with our 2020 data wave, and whose societal effects were still prominent during the 2022 wave. This rapid digitalization, which our findings in Section 3.1 empirically document, was substantively driven by public health policies enacted during the pandemic. For instance, the mandatory nationwide implementation of QR-based “health codes” (jiankangma) for accessing nearly all public spaces compelled digital adoption among all age groups, including older adults. This policy-driven shift occurred alongside significant disruptions to social capital and mental health, placing older adults in a situation of what has been described in the literature as a “double burden” of digital and social exclusion (Seifert et al., 2021). The pandemic also created a complex interplay between stress, internet use, and wellbeing during this period (Nimrod, 2020; Hwang et al., 2022). Therefore, the observed relationships throughout our study should be interpreted with the understanding that they occurred within this extraordinary global health crisis.

### 4.6 Policy implications

This investigation’s findings regarding suppression effects carry significant implications for public policies promoting digital inclusion among older adults. Policymakers must acknowledge digital technology’s dual impact and implement more nuanced intervention strategies:

First, transcend physical access to emphasize capability and trust. Policies merely providing devices and internet connectivity (addressing first-level divides) prove insufficient. As this investigation demonstrates, deficient skills and confidence (second- and third-level divides) precipitate negative social consequences. Policy focus must transition from simple “technological connection” to “meaningful and safe empowerment.”

Second, promote “safe bridging” as digital literacy education’s core. To mitigate BrSC erosion, digital literacy programs for older adults must exceed basic operational training. Curricula should center on enhancing cybersecurity awareness, fraud identification, and critical information assessment competencies. The fundamental objective involves helping older adults reconstruct generalized trust necessary for forming beneficial weak ties in the digital age.

Third, implement differentiated strategies leveraging strengths while mitigating weaknesses. Addressing suppression effects, policies should adopt dual approaches. They should continue supporting simple, user-friendly platforms (e.g., video chat applications) to maintain and amplify beneficial pathways strengthening BSC. Conversely, for complex platforms facilitating social network expansion but carrying elevated risks, comprehensive training centered on “safety” and “trust” must be provided to mitigate harmful pathways eroding BrSC.

### 4.7 Limitations and future research

This study contains several limitations, which indicate directions for future research. First, path analysis providing core evidence for our proposed mechanism utilized cross-sectional data, precluding complete elimination of reverse causality possibilities. Future research should employ longitudinal Structural Equation Modeling to more rigorously examine causal timing of the proposed suppression effect. Second, our BrSC measurement relied on proxy variables, potentially not capturing the theoretical construct’s complete meaning. Future studies could utilize qualitative methods, including in-depth interviews, to provide complementary evidence with enhanced depth and detail. Third, our study operationalized mental health via depressive symptoms. While this is a core indicator, future research could provide a more holistic view by including other dimensions, such as cognitive function or positive indicators of wellbeing. Fourth, our study is limited by the inability to directly model the COVID-19 pandemic’s impact due to data constraints within the CFPS. While our panel fixed-effects model controls for time-invariant individual traits, it cannot fully parse out the unique, time-varying effects of the pandemic from other secular trends. Future research should use event-study designs to disentangle its specific effects. Finally, although our PSM treatment group definition was theoretically grounded and validated through robustness checks, heterogeneity within this group warrants further exploration. Future research could design and test more nuanced digital inclusion intervention programs examining differential effects across various digital divide group types.

## 5 Conclusion

Employing a multi-method path decomposition strategy, this investigation elucidated the complex relationship between the digital divide and older adults’ mental health. The principal finding reveals a suppression effect: the ostensibly non-significant macroscopic relationship between these constructs results from mutual cancellation of direct and indirect pathways operating in opposing directions. Specifically, digital participation’s direct psychological benefits and BSC strengthening effects are substantially neutralized by indirect social risks generated through higher-order “motivational access” eroding BrSC. Older adults’ ultimate mental health status represents the net outcome of interplay between these opposing forces. Therefore, this study’s conclusion transcends simple “pros and cons” debates, emphasizing that future digital inclusion policies must transition from merely “bridging access” to “empowering trust.” This can be achieved through differentiated strategies enabling older adults to safely navigate the digital world’s dual nature, thereby realizing an inclusive and healthy digital aging society.

## Data Availability

Publicly available datasets were analyzed in this study. This data can be found at: http://www.isss.pku.edu.cn/cfps/index.htm.
