# Supplementary material for: The complex relationship between the digital divide, social capital, and mental health among older adults: a multi-method path decomposition
Source: Front Psychol. 2025 Nov 10;16:1670203. doi: 10.3389/fpsyg.2025.1670203 (PMC12640981; doi:10.3389/fpsyg.2025.1670203)
Supplement: Supplementary file 1 [file Supplementary_file_1.docx]

Supplementary Material

**Appendix** Full specification of the GSEM

$$\text{MH}_{i}=c_{1}^{'}\text{Physical\_Access}_{i}+c_{2}^{'}\text{Usage}_{i}+c_{3}^{'}{Motivational\_Access\_low}_{i}+c_{4}^{'}{Motivational\_Access\_high}_{i}+b_{1}{Neighbor\_Trust}_{i}+b_{2}\text{Child-parent\_Relationship}_{i}+b_{3}\text{Financial\_Transfers}_{i}+b_{4}\text{Contact\_Frequency}_{i}+b_{5}\text{Communication\_Frequency}_{i}+b_{6}\text{Trust\_in\_Strangers}_{i}+b_{7}\text{Interpersonal\_Relationships}_{i}+\Gamma_{1}^{'}Z_{i}+\zeta_{{MH}_{i}} \left( 1 \right)$$

$$\text{Neighbor\_Trust}_{i}=a_{1}\text{Physical\_Access}_{i}+a_{2}\text{Usage}_{i}+a_{3}\text{Motivational\_Access\_low}_{i}+a_{4}\text{Motivational\_Access\_high}_{i}+\Gamma_{2}^{'}Z_{i}+\zeta_{{Neighbor\_Trust}_{i}} \left( 2 \right)$$

$$\text{Child-parent\_Relationship}_{i}=a_{5}\text{Physical\_Access}_{i}+a_{6}\text{Usage}_{i}+a_{7}{Motivational\_Access\_low}_{i}+a_{8}\text{Motivational\_Access\_high}_{i}+\Gamma_{3}^{'}Z_{i}+\zeta_{\text{Child-parent\_Relationship}_{i}} \left( 3 \right)$$

$$\text{Financial\_Transfers}_{i}=a_{9}\text{Physical\_Access}_{i}+a_{10}\text{Usage}_{i}+a_{11}{Motivational\_Access\_low}_{i}+a_{12}\text{Motivational\_Access\_high}_{i}+\Gamma_{4}^{'}Z_{i}+\zeta_{\text{Financial\_Transfers}_{i}} \left( 4 \right)$$

$$\text{Contact\_Frequency}_{i}=a_{13}\text{Physical\_Access}_{i}+a_{14}\text{Usage}_{i}+a_{15}{Motivational\_Access\_low}_{i}+a_{16}\text{Motivational\_Access\_high}_{i}+\Gamma_{5}^{'}Z_{i}+\zeta_{\text{Contact\_Frequency}_{i}} \left( 5 \right)$$

$$\text{Communication\_Frequency}_{i}=a_{17}\text{Physical\_Access}_{i}+a_{18}\text{Usage}_{i}+a_{19}{Motivational\_Access\_low}_{i}+a_{20}\text{Motivational\_Access\_high}_{i}+\Gamma_{6}^{'}Z_{i}+\zeta_{\text{Communication\_Frequency}_{i}} \left( 6 \right)$$

$$\text{Trust\_in\_Strangers}_{i}=a_{21}\text{Physical\_Access}_{i}+a_{22}\text{Usage}_{i}+a_{23}{Motivational\_Access\_low}_{i}+a_{24}\text{Motivational\_Access\_high}_{i}+\Gamma_{7}^{'}Z_{i}+\zeta_{\text{Trust\_in\_Strangers}_{i}} \left( 7 \right)$$

$$\text{Interpersonal\_Relationships}_{i}=a_{25}\text{Physical\_Access}_{i}+a_{26}\text{Usage}_{i}+a_{27}{Motivational\_Access\_low}_{i}+a_{28}\text{Motivational\_Access\_high}_{i}+\Gamma_{8}^{'}Z_{i}+\zeta_{\text{Interpersonal\_Relationships}_{i}} \left( 8 \right)$$

In these equations, the three exogenous independent variables—$\text{Physical\_Access}_{i}$, $\text{Usage}_{i}$, and${\text{Motivational}\text{\_Access}}_{i}$,—are the observable dimensions of the digital divide. The five indicators of bonding social capital ($\text{Neighbor\_Trust}_{i}$, ${\text{Child-}\text{parent\_Relationship}}_{i}$, $\text{Financial\_Transfers}_{i}$, $\text{Contact\_Frequency}_{i}$, and $\text{Communication\_Frequency}_{i}$) and the two indicators of bridging social capital ($\text{Trust\_in\_Strangers}_{i}$ and $\text{Interpersonal\_Relationships}_{i}$) serve as observable mediators, and together they predict the final dependent variable, $\text{MH}_{i}$. $Z_{i}$ represents the vector of all exogenous control variables. The model simultaneously estimates the effects of the digital divide dimensions on social capital (path a), the effects of social capital on mental health (path b), and the direct effect of the digital divide after controlling for the mediators (path c').
