# Supplementary material for: The complex relationship between the digital divide, social capital, and mental health among older adults: a multi-method path decomposition
Source: Front Psychol. 2025 Nov 10;16:1670203. doi: 10.3389/fpsyg.2025.1670203 (PMC12640981; doi:10.3389/fpsyg.2025.1670203)
Supplement: Supplementary file 2 [file Table_1.docx]

Supplementary Material

**Table S1** Robustness check: ATT estimation within the internet user subsample

| **Matching Method** | **ATT** | **Std. Err.** | **t/z-statistic** | **P-value** |
| --- | --- | --- | --- | --- |
| 1:1 Nearest Neighbor | 0.07 | 0.25 | 0.30 | > 0.1 |
| *Bootstrap SE* | 0.00 | 0.39 | 0.00 | 1.00 |
| 1:4 Nearest Neighbor | 0.01 | 0.21 | 0.05 | > 0.1 |
| *Bootstrap SE* | 0.16 | 0.33 | 0.48 | 0.63 |
| Caliper Matching (0.25 SD) | 0.07 | 0.25 | 0.30 | > 0.1 |
| *Bootstrap SE* | 0.20 | 0.38 | 0.51 | 0.61 |
| Kernel Matching | 0.17 | 0.19 | 0.89 | > 0.1 |
| *Bootstrap SE* | -0.34 | 0.26 | -1.32 | 0.19 |

This analysis was conducted exclusively on the internet user subsample, comparing "low-efficiency users" (treatment group) with "high-efficiency users" (control group). Rows in italics represent the standard errors and corresponding z/p-values obtained using the bootstrap method (500 repetitions)

**Table S2** Rosenbaum bounds sensitivity analysis for the 1-to-4 nearest neighbor matching

| **Gamma** | **sig+** | **sig-** | **t-hat+** | **t-hat-** | **CI+** | **CI-** |
| --- | --- | --- | --- | --- | --- | --- |
| 1 | 0 | 0 | 5.5 | 5.5 | 5.5 | 5.5 |
| 1.1 | 0 | 0 | 5.5 | 5.5 | 5 | 6 |
| 1.2 | 0 | 0 | 5 | 6 | 5 | 6 |
| 1.3 | 0 | 0 | 5 | 6 | 5 | 6 |
| 1.4 | 0 | 0 | 5 | 6 | 4.5 | 6.5 |
| 1.5 | 0 | 0 | 4.5 | 6.5 | 4.5 | 6.5 |
| 1.6 | 0 | 0 | 4.5 | 6.5 | 4.5 | 6.5 |
| 1.7 | 0 | 0 | 4.5 | 6.5 | 4.5 | 7 |
| 1.8 | 0 | 0 | 4.5 | 6.5 | 4 | 7 |
| 1.9 | 0 | 0 | 4 | 7 | 4 | 7 |
| 2 | 0 | 0 | 4 | 7 | 4 | 7 |

**Table S3** Covariate balance tests for the PSM analysis by gender and residence

| **Group** | **Subgroup** | **Matching method** | **Sample** | **Ps R2** | **LR chi2** | **p>chi2** | **Mean Bias** | **Med Bias** | **Rubin's B** | **Rubin's R** | **%Var** |
| --- | --- | --- | --- | --- | --- | --- | --- | --- | --- | --- | --- |
| **Gender** | Female | 1:1 Nearest Neighbor | Unmatched | 0.177 | 410.14 | <0.001 | 21.3 | 12.9 | 110.1* | 1.1 | 65 |
|  |  |  | Matched | 0.027 | 106.22 | <0.001 | 8.2 | 9.4 | 39.2* | 1.21 | 47 |
|  |  | Kernel Matching | Unmatched | 0.177 | 410.14 | <0.001 | 21.3 | 12.9 | 110.1* | 1.1 | 65 |
|  |  |  | Matched | 0.023 | 88.71 | <0.001 | 7.1 | 6.1 | 35.8* | 1.24 | 35 |
|  | Male | 1:1 Nearest Neighbor | Unmatched | 0.141 | 380.87 | <0.001 | 21.2 | 21.1 | 95.7* | 1.37 | 65 |
|  |  |  | Matched | 0.025 | 105.59 | <0.001 | 6.2 | 5.9 | 37.2* | 1.44 | 18 |
|  |  | Kernel Matching | Unmatched | 0.141 | 380.87 | <0.001 | 21.2 | 21.1 | 95.7* | 1.37 | 65 |
|  |  |  | Matched | 0.018 | 76.23 | <0.001 | 6.1 | 5.5 | 31.6* | 1.32 | 18 |
| **Residence** | Rural | 1:1 Nearest Neighbor | Unmatched | 0.138 | 300.86 | <0.001 | 19.8 | 17.4 | 97.6* | 1.23 | 71 |
|  |  |  | Matched | 0.027 | 124.02 | <0.001 | 7.3 | 5.5 | 38.6* | 0.83 | 47 |
|  |  | Kernel Matching | Unmatched | 0.138 | 300.86 | <0.001 | 19.8 | 17.4 | 97.6* | 1.23 | 71 |
|  |  |  | Matched | 0.013 | 60.47 | <0.001 | 4.8 | 3.6 | 26.9* | 1.39 | 24 |
|  | Urban | 1:1 Nearest Neighbor | Unmatched | 0.137 | 373.41 | <0.001 | 18.5 | 16.5 | 92.8* | 1.27 | 59 |
|  |  |  | Matched | 0.011 | 41.92 | 0.006 | 5.4 | 4.2 | 24.6 | 1.04 | 24 |
|  |  | Kernel Matching | Unmatched | 0.137 | 373.41 | <0.001 | 18.5 | 16.5 | 92.8* | 1.27 | 59 |
|  |  |  | Matched | 0.013 | 49.79 | 0.001 | 5.1 | 4.2 | 26.8* | 1.32 | 12 |
